# Ocular Phenotypes and Novel *SLC45A2* Variants in Patients with Oculocutaneous Albinism Type 4

**DOI:** 10.3390/genes17080963

**Published:** 2026-08-17

**Authors:** Chonglin Chen, Bingqi Wang, Ye Zheng, Junyi Liu, Xinping Yu

**Affiliations:** State Key Laboratory of Ophthalmology, Zhongshan Ophthalmic Center, Sun Yat-sen University, Guangdong Provincial Key Laboratory of Ophthalmology and Visual Science, Guangzhou 510060, China; chenchonglin@gzzoc.com (C.C.); weirdocuddle@163.com (B.W.); zhengy55@126.com (Y.Z.); ljyyi123@163.com (J.L.)

**Keywords:** oculocutaneous albinism type 4, ocular characteristics, novel variants

## Abstract

Objectives: *SLC45A2*-related oculocutaneous albinism type 4 (OCA4) is a genetically defined subtype of albinism; however, its ocular phenotype remains incompletely characterized. This study aimed to describe the systemic, ophthalmic, and genetic features of patients with genetically confirmed *SLC45A2*-related OCA4. Methods: Ninety patients with clinically diagnosed albinism were enrolled and underwent genetic testing. Patients with genetically confirmed *SLC45A2*-related OCA4 were included for further analysis. Demographic information, systemic pigmentation features, ophthalmic findings, and *SLC45A2* variants were analyzed. Results: Five unrelated patients with genetically confirmed *SLC45A2*-related OCA4 were included, aged 5–33 years. Best-corrected visual acuity ranged from 0.1 to 1.0 logMAR, and spherical equivalent refractive error varied widely from −7.25 to +7.63 D. All patients exhibited iris transillumination defects and moderate-to-severe fundus hypopigmentation, both graded 2–3. Foveal hypoplasia was universally present, ranging from grade 1 to grade 4, with three patients showing grade 4 hypoplasia. Genetic analysis revealed nine disease-associated alleles representing eight distinct *SLC45A2* variants: seven missense variants and one splice-altering variant. One patient carried a homozygous variant, and four carried compound heterozygous variants. Three novel variants were identified: c.1459C>G (p.Gln487Glu), c.137A>G (p.Glu46Gly), and c.561A>G. Conclusions: This study characterizes the ocular phenotype of *SLC45A2*-related OCA4, including iris transillumination defects, fundus hypopigmentation, and foveal hypoplasia of varying severity. Three novel *SLC45A2* variants were identified, expanding the mutational spectrum of OCA4.

## 1. Introduction

Albinism comprises a genetically heterogeneous group of inherited disorders characterized by reduced or absent melanin biosynthesis, typically affecting the skin, hair, and eyes [1]. Ocular involvement is often prominent and may include reduced visual acuity, nystagmus, foveal hypoplasia, iris transillumination, fundus hypopigmentation, refractive errors, and abnormal decussation of the optic pathways [2,3]. These visual abnormalities can substantially impair visual development and quality of life, highlighting the importance of detailed ophthalmic characterization in the clinical evaluation of individuals with albinism. The prevalence of oculocutaneous albinism varies among populations, with an estimated frequency of approximately 1 in 10,000–20,000 individuals worldwide [4]. The contribution of *SLC45A2* variants to OCA varies among populations, with a relatively higher frequency reported in some Asian populations compared with other ethnic groups [5].

Among the genes implicated in oculocutaneous albinism (OCA), *SLC45A2* is recognized as the causative gene for oculocutaneous albinism type 4 (OCA4). It encodes a multi-pass membrane-associated transporter protein predominantly expressed in melanocytes and is thought to regulate melanosomal homeostasis, including ionic and pH balance, thereby supporting normal melanin synthesis [6,7,8,9]. Pathogenic variants in *SLC45A2* may impair melanosomal function, disrupt pigmentation pathways, and give rise to variable systemic and ocular phenotypes.

The clinical manifestations of albinism vary widely, and the severity of ocular involvement does not necessarily correlate with the degree of cutaneous or systemic hypopigmentation. Although previous reports have suggested that patients with OCA4 may present with variable pigmentation and diverse clinical features [10], detailed ophthalmic descriptions of genetically confirmed cases remain limited. In the present study, we report five unrelated patients with genetically confirmed *SLC45A2*-related OCA4 identified from a cohort of 90 individuals with albinism. We describe their systemic pigmentation features, comprehensive ocular findings, and associated *SLC45A2* variants. These findings expand the known mutation spectrum of OCA4 and provide supporting evidence for the ocular phenotype associated with this subtype.

## 2. Patients and Methods

### 2.1. Study Subjects and Clinical Evaluation

This study was approved by the Institutional Review Board of Zhongshan Ophthalmic Center, Sun Yat-sen University (approval number: 2022KYPJ245), and conducted in accordance with the Declaration of Helsinki and the approved study protocol. Written informed consent was obtained from the patients or their legal guardians for clinical examinations, genetic testing, and the publication of de-identified data and images. The diagnosis of OCA4 was established based on characteristic clinical manifestations and confirmed by molecular genetic testing demonstrating biallelic variants in *SLC45A2*.

### 2.2. Ophthalmic Examinations

All patients underwent comprehensive ophthalmic evaluation, including best-corrected visual acuity (BCVA), cycloplegic or manifest refraction, ocular alignment assessment, slit-lamp biomicroscopy, fundus examination, and optical coherence tomography (OCT). Additional clinical information, including age at presentation, sex, family history, and extraocular pigmentation features, was collected.

BCVA was measured using a standard logarithmic visual acuity chart and converted to the logarithm of the minimum angle of resolution (logMAR) units for descriptive comparison when appropriate. Refractive status was recorded as spherical equivalent refraction. Nystagmus characteristics, including the presence and type of nystagmus, were documented based on clinical examination. Strabismus was assessed by cover/uncover and prism alternate cover testing when feasible. Anterior segment findings were evaluated by slit-lamp examination, with particular attention to iris transillumination and lens abnormalities. Iris translucency was graded from 0 to 4 as described by Kruijt et al. [11]. Fundus pigmentation was assessed by dilated fundus examination and color fundus photography when available (Carl Zeiss Meditec AG), and categorized according to the degree of retinal hypopigmentation [12]. Macular imaging was performed using an OCT system (Carl Zeiss AG, Jena, Germany). Foveal hypoplasia was graded according to the structural grading system proposed by Thomas et al. [12]., which classifies foveal hypoplasia based on the stage at which normal foveal development is arrested on OCT.

### 2.3. Genetic Analysis

Peripheral blood samples were obtained from the probands and, when available, from their parents and other family members. Genomic DNA was extracted from peripheral blood leukocytes using standard protocols. Whole-exome sequencing was performed for molecular diagnosis, followed by bioinformatic filtering and annotation with a focus on genes associated with albinism, ocular hypopigmentation, and inherited ocular disorders.

The exome was captured using the Twist HumanCore Exome Kit (Twist Bioscience, South San Francisco, CA, USA) and sequenced in paired-end 2 × 150 bp format on the Illumina NovaSeq 6000 platform. Read alignment to the human reference genome GRCh38 and variant calling were performed using the Sentieon Genomics DNASeq software pipeline (Sentieon, Inc., San Jose, CA, USA), and variant annotation was performed using ANNOVAR (https://annovar.openbioinformatics.org, accessed on 15 June 2026). Variants were prioritized according to allele frequency, predicted functional consequence, inheritance pattern, and known gene–disease associations. Particular attention was given to rare, protein-altering variants in *SLC45A2*. Candidate variants identified by whole-exome sequencing were confirmed by Sanger sequencing. When DNA samples from family members were available, segregation analysis was performed to determine the parental origin of the variants, assess whether compound heterozygous variants were present in trans, and confirm autosomal recessive inheritance where possible.

Variant nomenclature was described according to the recommendations of the Human Genome Variation Society (HGVS), based on the *SLC45A2* reference transcript NM_016180.5. The pathogenicity of the identified variants was assessed by integrating evidence from population databases, disease-associated variant databases, computational prediction tools, and the available clinical phenotype. Population frequency was evaluated using databases such as gnomAD [13]. Previously reported disease associations were searched in ClinVar [14], the Human Gene Mutation Database [15], and the published literature. The potential functional impact of missense or splice-region variants was assessed using in silico prediction tools, including PolyPhen-2 [16] (https://genetics.bwh.harvard.edu/pph2, accessed on 15 June 2026), MutationTaster [17] (https://www.mutationtaster.org, accessed on 15 June 2026), and REVEL. Variants were classified according to the American College of Medical Genetics and Genomics/Association for Molecular Pathology (ACMG/AMP) guidelines as pathogenic, likely pathogenic, variant of uncertain significance, likely benign, or benign [18]. Novel variants were defined as variants that were absent from the consulted population databases and disease-associated variant databases and had not been reported in the published literature at the time of analysis. The potential splicing effect of the synonymous variant c.561A>G was assessed using SpliceAI, as implemented in GeneBe (https://genebe.net, accessed on 15 June 2026).

## 3. Results

### 3.1. Demographic Characteristics and Systemic Features

Among the 90 patients with albinism enrolled in our cohort, five unrelated individuals were identified as having genetically confirmed *SLC45A2*-related oculocutaneous albinism type 4 (OCA4). The cohort comprised three females and two males, ranging in age from 5 to 33 years. No patient reported a family history of albinism. All affected individuals exhibited generalized hypopigmentation, characterized by fair skin and light-colored hair ranging from pale yellow to blond. No extra-systemic manifestations were reported in any patient. The demographic characteristics and systemic pigmentation features of these patients are summarized in Table 1.

### 3.2. Ocular Characteristics

**Figure 1:**
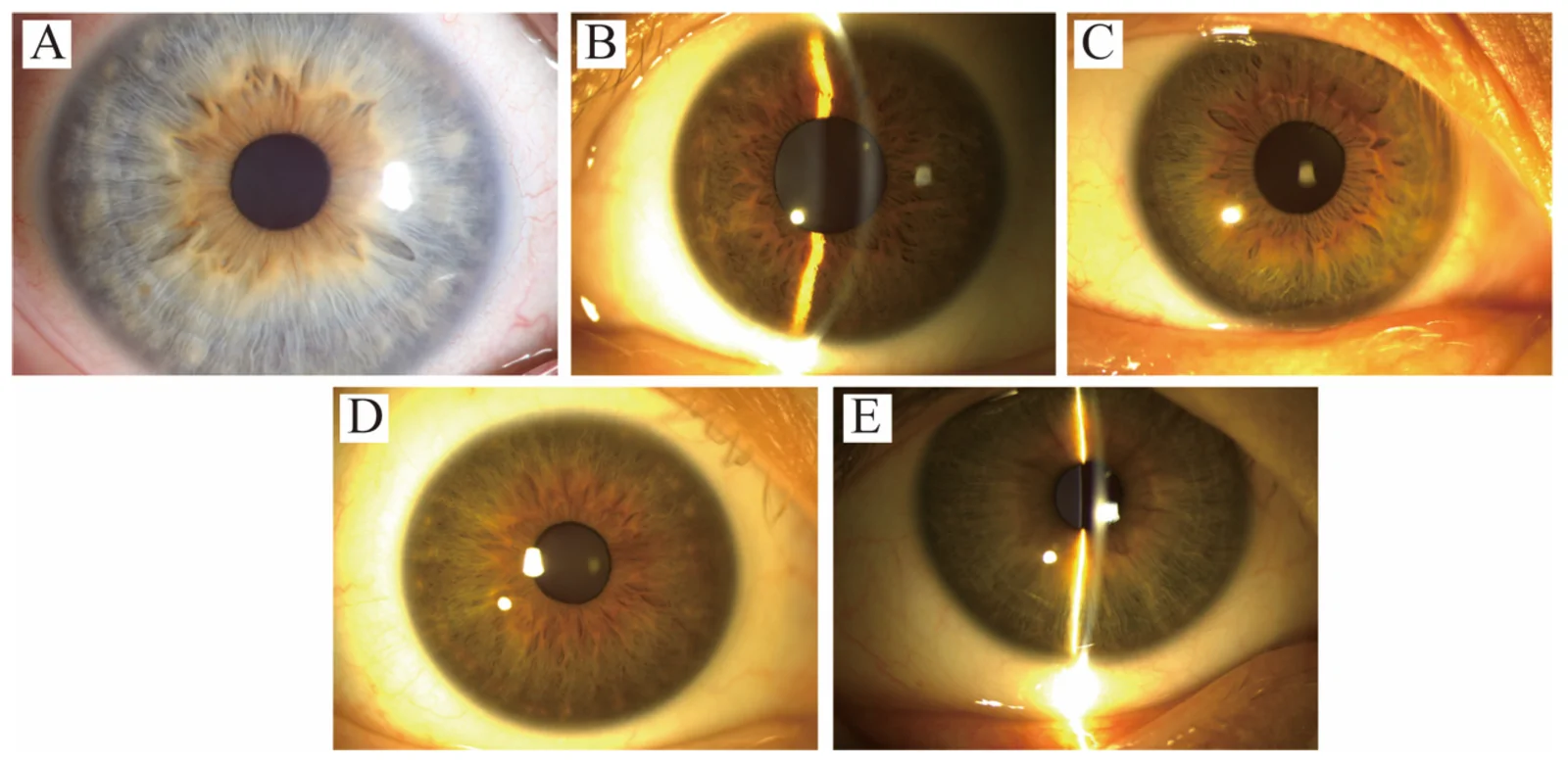
Representative anterior segment photographs of patients with OCA4. (**A**) Patient A002 exhibited a gray iris with central annular light-brown pigmentation and grade 3 iris transillumination. (**B**) Patient A010 exhibited a dark-brown iris with grade 2 iris transillumination. (**C**) Patient A024 and (**D**) Patient A034 exhibited light-brown irises with central annular dark-brown pigmentation and grade 2 iris transillumination. (**E**) Patient A035 exhibited a gray iris with central annular light-brown pigmentation and grade 3 iris transillumination. Grade 2 and grade 3 transillumination indicate diffuse iris translucency and circumferential lens visibility, respectively. (All anterior segment photographs shown in this figure represent the right eyes of the corresponding patients).

**Figure 2:**
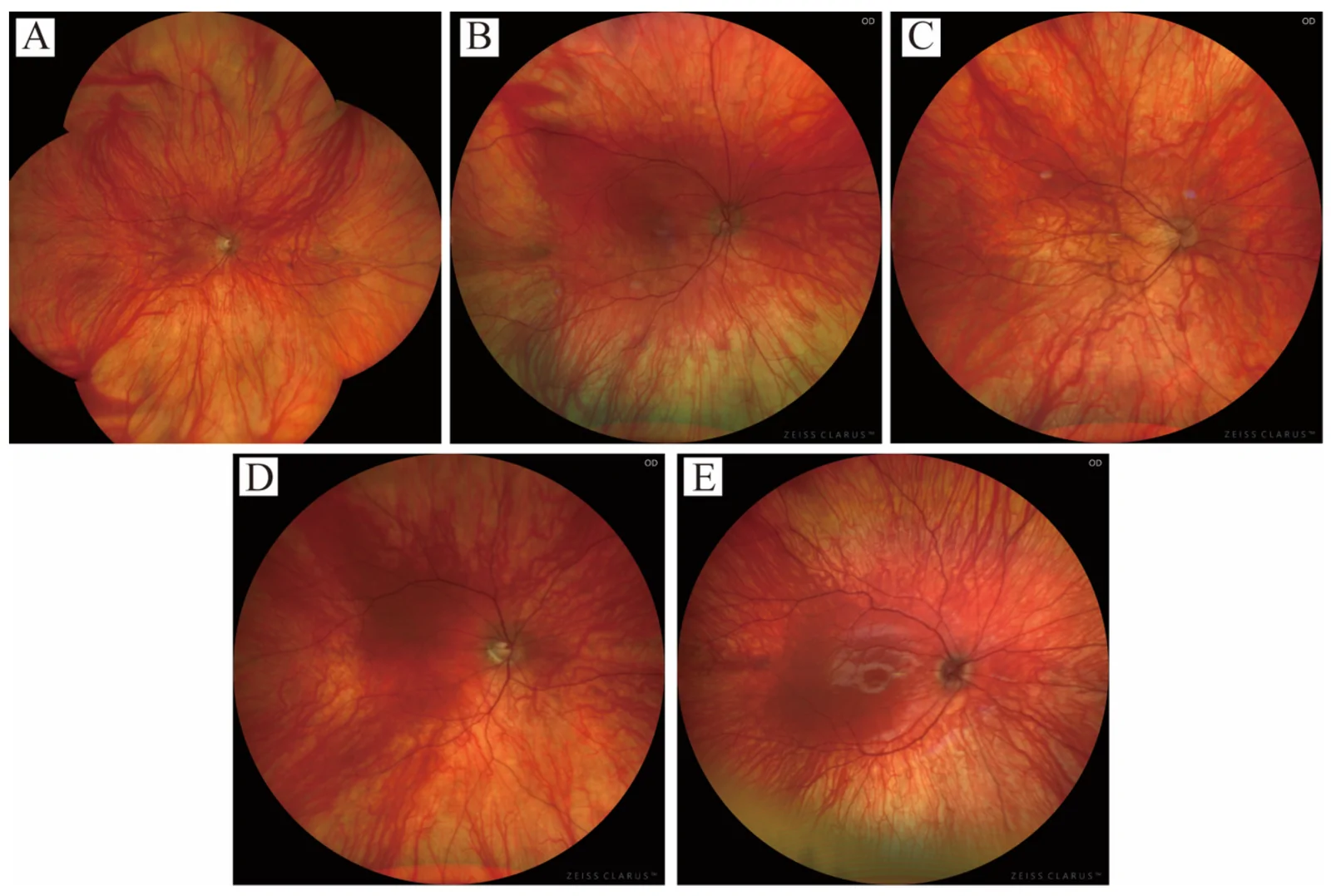
Representative fundus photographs of patients with OCA4. (**A**) Patient A002 showed grade 3 fundus hypopigmentation. (**B**) Patient A010 showed grade 2 fundus hypopigmentation. (**C**) Patient A024 showed grade 3 fundus hypopigmentation. (**D**) Patient A034 and (**E**) patient A035 showed grade 2 fundus hypopigmentation.

**Figure 3:**
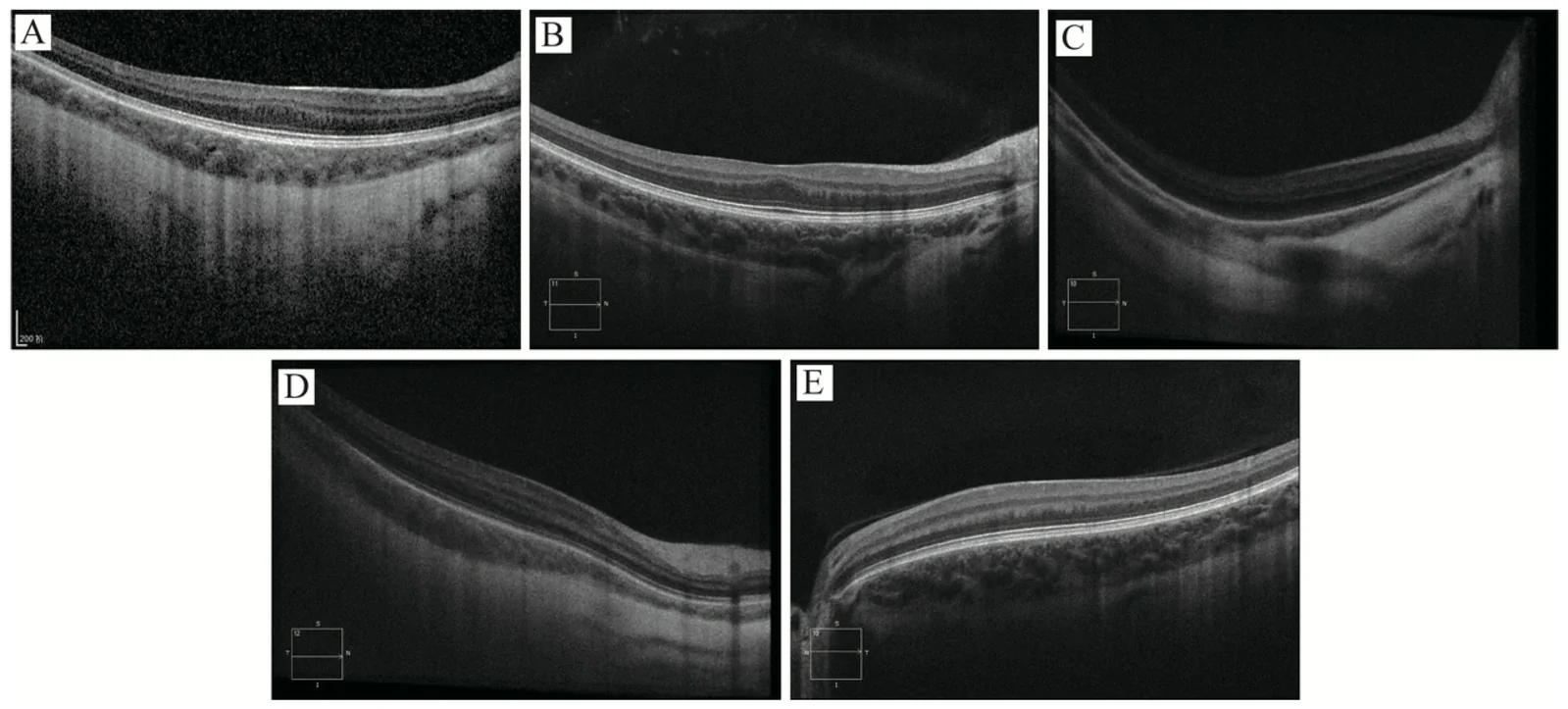
Optical coherence tomography images showing macular morphology in patients with OCA4. (**A**) Patient A002 showed grade 3 foveal hypoplasia in the right eye. (**B**) Patient A010 showed grade 1 foveal hypoplasia in the right eye. (**C**) Patient A024 and (**D**) Patient A034 showed grade 4 foveal hypoplasia in the right eyes. (**E**) Patient A035 showed grade 4 foveal hypoplasia in the left eye.

The ophthalmic characteristics of the five patients with *SLC45A2*-related OCA4 are summarized in Table 2. Best-corrected visual acuity (BCVA) ranged from 0.1 to 1.0 logMAR. Refractive status was heterogeneous, spanning high myopia to high hyperopia, with spherical equivalent values ranging from −7.25 D to +7.63 D and axial lengths ranging from 20.38 to 25.99 mm. Photophobia was present in all patients, while nystagmus was observed in four of five patients (80%). Strabismus was identified in two patients, including one case each of esotropia and exotropia. Iris transillumination defects were observed in all patients, with grades ranging from 2 to 3 (Figure 1). Fundus hypopigmentation was consistently present and graded as moderate to severe (grades 2–3) (Figure 2). Foveal hypoplasia was detected in all patients, with severity ranging from grade 1 to grade 4, and three patients exhibited grade 4 hypoplasia (Figure 3).

Grade 3 was defined as visible choroidal vessels in the macular region, whereas grade 2 was defined as visible choroidal vessels in the posterior pole with macular sparing. (All fundus photographs shown in this figure represent the right eyes of the corresponding patients).

Grade 1 was characterized by an abnormal but present foveal pit, extrusion of the inner retinal layers, outer segment layer lengthening, and outer nuclear layer widening. Grade 2 lacked a foveal pit. Grade 3 additionally lacked outer segment layer lengthening. Grade 4, the most severe grade, also lacked outer nuclear layer widening.

### 3.3. Genetic Findings

Genetic analysis identified nine disease-associated variant alleles corresponding to eight distinct *SLC45A2* variants in five unrelated patients with OCA4 (Table 3). One patient (A002) carried a homozygous *SLC45A2* variant, whereas the remaining four patients harbored compound heterozygous variants, consistent with the autosomal recessive inheritance pattern of OCA4. The identified variants comprised seven missense variants and one splice-site variant. Among the eight distinct variants, three variants, c.1459C>G (p.Gln487Glu), c.137A>G (p.Glu46Gly), and c.561A>G, were novel. The novel synonymous variant c.561A>G was predicted to potentially affect pre-mRNA splicing. dbscSNV yielded ADA and RF scores of 1.00 and 0.94, respectively, both supporting a splice-altering effect. Consistently, SpliceAI showed a maximum delta score of 0.51 for donor loss (DS_DL = 0.51; position offset = −1), suggesting a potential disruption of normal donor-site recognition. Segregation analysis demonstrated that c.561A>G was inherited from the mother, whereas variant c.137A>G was inherited from the father, confirming that the two variants were in trans. Detailed molecular characteristics of the identified variants are summarized in Table 3.

**Figure 4:**
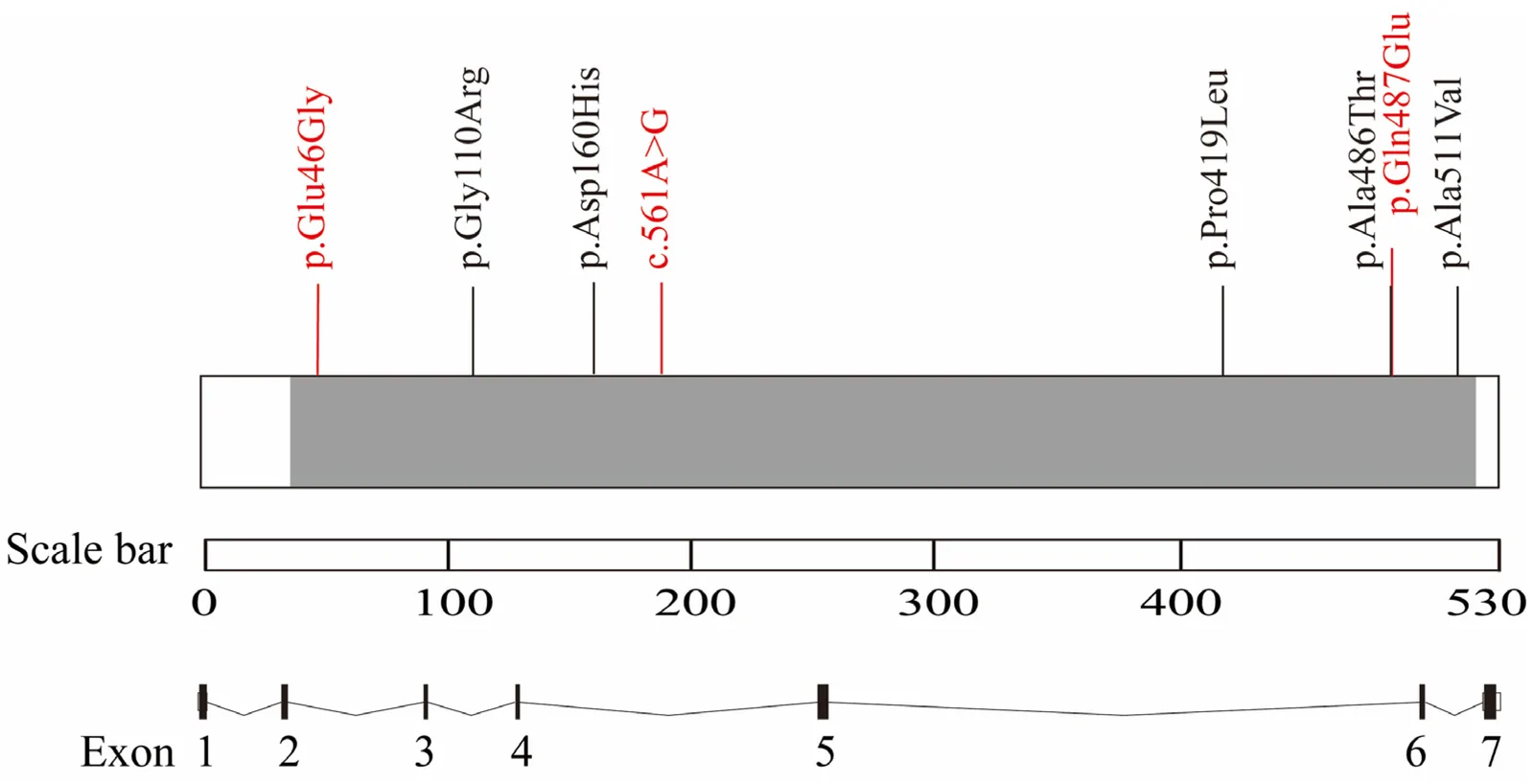
Schematic representation of the SLC45A2 protein domain, gene structure, and identified variants. Novel variants identified in this study are shown in red, whereas previously reported variants are shown in black.

The identified *SLC45A2* variants in our cohort were distributed across multiple coding exons of the 530-amino-acid melanosomal membrane-associated transporter. The missense variants p.Glu46Gly, p.Gly110Arg, p.Asp160His, p.Pro419Leu, p.Ala486Thr, p.Gln487Glu, and p.Ala511Val were located in different predicted topological regions of SLC45A2, including transmembrane helices and adjacent intra- or extracellular regions (Figure 4).

The upper panel shows the schematic structure of the human SLC45A2 protein, which consists of 530 amino acids. The MFS_SLC45_SUC conserved domain spans amino acids 35–521 and covers most of this multi-pass transporter, as indicated by the gray region. The scale bar denotes amino acid positions. The lower panel shows the exon–intron organization of the SLC45A2 gene, with exon numbers indicated. Variants identified in this study and those previously reported are annotated above the protein schematic, and novel variants identified in the present study are highlighted in red.

## 4. Discussion

OCA4 is a subtype of nonsyndromic oculocutaneous albinism associated with pathogenic variants in *SLC45A2*. Its estimated worldwide prevalence is approximately 1:100,000 [22]; however, its proportion among patients with albinism varies markedly across populations. In European and North American cohorts, OCA4 generally accounts for a relatively small fraction of OCA cases, including approximately 4% in a Dutch cohort and around 6% in non-Hispanic Caucasian patients [23,24]. By contrast, OCA4 is substantially more common in East Asian populations, particularly in Japan, where it accounts for approximately 25–30% of molecularly confirmed OCA cases [5]. In previous Chinese cohorts, *SLC45A2*-related OCA has been reported in approximately 10–13% of OCA cases [25]. In the present Chinese cohort, five of 90 patients with albinism were found to harbor biallelic variants in *SLC45A2*, accounting for 5.6% of all cases.

*SLC45A2*-related OCA4 is classically inherited in an autosomal recessive manner, with affected individuals typically carrying biallelic variants in either a homozygous or compound heterozygous state. The mutational spectrum of *SLC45A2* is heterogeneous and includes missense, nonsense, splice-site, frameshift, small insertion/deletion, promoter, and larger deletion/duplication variants; however, missense variants appear to be the most frequently reported type [22]. This is also reflected by several recurrent population-specific missense alleles, such as p.Asp157Asn, which is commonly reported in Japanese and Korean patients [26], and p.Asp160His, which has been described as a common allele in Chinese patients with OCA4 [25].

According to the CDD annotation integrated in GeneCards, human SLC45A2 contains an MFS_SLC45_SUC conserved domain spanning amino acids 35–521, covering most of the 530-amino-acid multi-pass transporter [27]. The missense variants identified in this study, including p.Glu46Gly, p.Gly110Arg, p.Asp160His, p.Pro419Leu, p.Ala486Thr, p.Gln487Glu, and p.Ala511Val, were all located within this conserved domain, with several mapping to regions predicted to correspond to, or lie close to, transmembrane segments. Given the importance of conserved transporter domains and membrane-spanning regions for protein conformation, membrane localization, and transport-related activity, these substitutions may impair SLC45A2 function. In contrast, c.561A>G (p.Thr187=), although synonymous at the amino acid level, was predicted to alter splicing, suggesting a potential pathogenic mechanism through abnormal pre-mRNA processing. These interpretations are based on domain annotation and in silico predictions, and further RNA- and protein-level studies are needed for functional validation.

This study has several limitations. First, the sample size of genetically confirmed *SLC45A2*-related OCA4 was small, reflecting the rarity of this subtype and limiting robust genotype–phenotype correlation analysis. Second, the pathogenic effects of the identified variants, particularly the novel variants, were inferred mainly from clinical and in silico evidence without functional validation. Finally, this was a single-center Chinese cohort, and the findings may not be fully generalizable to other populations.

## 5. Conclusions

In conclusion, this study characterized the clinical and genetic features of five unrelated Chinese patients with *SLC45A2*-related OCA4. All patients showed characteristic manifestations of nonsyndromic oculocutaneous albinism, including generalized hypopigmentation, iris transillumination defects, fundus hypopigmentation, and foveal hypoplasia, with variable degrees of ocular involvement. Eight distinct *SLC45A2* variants were identified, including three novel variants, thereby expanding the mutational spectrum of *SLC45A2* in Chinese patients. The localization of most missense variants within the conserved transporter domain supports their potential functional relevance. These findings further underscore the phenotypic and genetic heterogeneity of OCA4 and highlight the importance of comprehensive molecular diagnosis in patients with albinism.

## Figures and Tables

**Table 1:** Demographic Characteristics and Systemic Pigmentation Features of Patients with *SLC45A2*-Related OCA4.

Patient ID	Sex	Age, Years	Family History	Hair Color	Skin Color
A002	F	32	None	Blond	White
A010	F	33	None	Blond	White
A024	M	31	None	Pale yellow	White
A034	M	30	None	Pale yellow	White
A035	F	5	None	Blond	White

**Table 2:** Ocular Characteristics of Patients with *SLC45A2*-Related OCA4.

Patient ID	BCVA, LogMAR (OD; OS)	Spherical Equivalent, D (OD; OS)	Axial Length, mm (OD;OS)	Photophobia	Nystagmus	Strabismus	Iris Transillumination Grade	Fundus Hypopigmentation Grade	Foveal Hypoplasia Grade
A002	0.4; 0.3	−2.87; −0.63	25.66; 24.87	Present	Present	Esotropia	3	3	3
A010	0.1; 0.2	−1.25; +0.38	23.5; 22.88	Present	Absent	None	2	2	1
A024	0.7; 0.5	−7.25; −3.63	25.48; 25.99	Present	Present	None	2	3	4
A034	0.7; 1.0	−5.13; −6.25	24.63; 25.2	Present	Present	Exotropia	2	2	4
A035	0.7; 1.0	+7; +7.63	20.77; 20.38	Present	Present	None	3	2	4

**Table 3:** Variants identified in *SLC45A2*.

Patient ID	Genomic Position (GRCh38)	cDNA Change	Protein Change	Variant Consequence	East Asian Allele Frequency	ACMG/AMP Criteria	ACMG Classification	MutationTaster Prediction	PolyPhen-2 Prediction	REVEL Score	Source
**A002**	chr5:33982320	c.478G>C	p.Asp160His	missense	-	PM1 + PM2 + PP3	Likely pathogenic	Disease-causing	Probably damaging	0.91	[19]
**A010**	chr5:33944782	c.1459C>G	p.Gln487Glu	missense	-	PM1 + PM2 + PM3 + PP3	Likely pathogenic	Disease-causing	Probably damaging	0.83	Novel
	chr5:33947275	c.1256C>T	p.Pro419Leu	missense	0.0000252	PM1 + PM2 + PP3	Pathogenic	Disease-causing	Probably damaging	0.85	[20]
**A024**	chr5:33944785	c.1456G>A	p.Ala486Thr	missense	-	PM1 + PM2 + PM5 + PP3	Pathogenic	Disease-causing	Probably damaging	0.84	[21]
	chr5:33944709	c.1532C>T	p.Ala511Val	missense	-	PM1 + PM2 + PM5	Likely pathogenic	Disease-causing	Probably damaging	0.61	[10]
**A034**	chr5:33982320	c.478G>C	p.Asp160His	missense	-	PM1 + PM2 + PP3	Likely pathogenic	Disease-causing	Probably damaging	0.91	[19]
	chr5:33984256	c.328G>A	p.Gly110Arg	missense	-	PM1 + PM2 + PP3	Likely pathogenic	Disease-causing	Probably damaging	0.73	[19]
**A035**	chr5:33984447	c.137A>G	p.Glu46Gly	missense	0.000164	PM1 + PM2 + PM3 + PP3	Likely pathogenic	Disease-causing	Probably damaging	0.95	Novel
	chr5:33982237	c.561A>G	p.Thr187=	synonymous	-	PM2 + PP3	Uncertain significance	-	-	-	Novel

## Data Availability

The data that support the findings of this study are available from the corresponding author upon reasonable request. The data are not publicly available because they contain information that could compromise the privacy of the research participants.
